# Epstein Barr Virus Infection Affects Function of Cytotoxic T Lymphocytes in Patients with Severe Aplastic Anemia

**DOI:** 10.1155/2018/6413815

**Published:** 2018-05-14

**Authors:** Tian Zhang, Chunyan Liu, Hui Liu, Lijuan Li, Ting Wang, Rong Fu

**Affiliations:** Department of Hematology, General Hospital of Tianjin Medical University, Tianjin, China

## Abstract

Severe aplastic anemia (SAA) is characterized by pancytopenia and failure of hematopoietic function and is caused by excessive functioning of cytotoxic T lymphocytes (CTLs). EBNA-1, a nucleoprotein of the Epstein Barr virus (EBV), can influence the proliferation and function of lymphocytes. We therefore tested the number of EBV copies in the CD8+ T cells of 27 patients with SAA and 10 healthy control subjects and observed the influences of EBNA-1 upon the CD8+ T cells of patients with SAA. The results showed that more EBV copies were found in the CD8+ T cells of patients with untreated SAA than in patients with SAA in remission or in the healthy control subjects. Their copy number was positively correlated with the expression of granzyme B and perforin, the secretion level of interferon-*γ* in CD8+ T cells, and the viability of CD8+ T cells, whereas no correlation was seen between the copy number and the interleukin 4 secretion level or the apoptosis rate. Meanwhile, the expression of granzyme B and perforin was reduced after EBNA-1 gene knockdown, whereas the interferon-*γ* secretion level and cell viability declined. Therefore, we infer that EBV infection may be a factor in the activation of CTLs and in damaging the bone marrow hematopoietic function of patients with SAA.

## 1. Introduction

Severe aplastic anemia (SAA) is characterized by severe pancytopenia, failure of hematopoietic function, an acute onset, rapid progression, and a high fatality rate [1]. SAA is an autoimmune disease whose immunotolerance mechanism has been shown to be broken and mediated by hyperfunctional T lymphocytes. Proliferation and over activation of cytotoxic T lymphocytes (CTLs; CD8+ T cells) are the direct causes of bone marrow failure in patients with SAA [2]. Our group conducted a series of studies in which we found abnormal expression of various immunomodulatory molecules on the surface of CTLs and an increased number of activated effective T cells (CD8+ human leukocyte antigen D related + T cells), with obviously higher levels of intracellular molecules, including perforin, granzyme B, tumor necrosis factor (TNF) *β*, and FasL, which confirmed that CTLs may be key cells in the pathogenesis of SAA [3–5]. A variety of viruses are known to lead to hyperfunction of CTLs via disruption of the immune balance and ultimately contribute to the occurrence of SAA. Hepatitis B is the most common of these viruses [6].

Epstein Barr virus (EBV) is a type of human T-lymphotropic double-stranded DNA virus that belongs to the herpes virus family, which was first isolated from African children with Burkitt lymphoma in 1964 by Epstein et al. [7]. EBV has a dual infection strategy: lytic infection and latent infection [8]. The CTLs' response plays an important role in fighting EBV infection, especially in persistent virus infection. In early stages of infectious mononucleosis, the non-EBV-specific CTLs in the peripheral blood are activated and play a part in the nonspecific immune attack. The infectious bodies then produce EBV-specific active CTLs, inhibiting the proliferation of EBV infected cells and preventing the recurrence of this infection [9]. EBNA-1 is a kind of nucleoprotein encoded by the BKRF1 genome. EBV has various stages of latency with varying viral gene expression, and EBNA1 is expressed during most of these stages. It can combine with the replication origin of EBV (oriP), which further plays a pivotal role in the replication of EBV DNA, the maintenance of EBV plasmid stabilization, and the transcription activation of EBV. In addition, the presence of EBNA-1 protein helps EBV escape the immune attack of T lymphocytes [10]. In recent years, several scholars have found that EBNA-1 has an influence on multiple cellular pathways, thus affecting the proliferation and function of the infected cells [11].

In recent years, researchers have authenticated that EBV infection can cause an immune imbalance, finally resulting in the onset of various autoimmune diseases, such as systemic lupus erythematosus and multiple sclerosis, and EBV infection has also been reported to cause SAA [12, 13], but its mechanism remains unclear. In a retrospective study of EBV infection in 43 treatment-naive patients with SAA from March 2014 to March 2017, we found that 21 patients (48.83%) with SAA had a previous EBV infection, whereas two patients with SAA had a current infection (4.65%). We propose that EBV can affect the function of T cells in patients with SAA. We therefore tested the number of BamH1W DNA sequence copies of the EBNA-1 encoding gene in the CD8+ T cells of 27 SAA patients and 10 healthy control subjects with a polymerase chain reaction- (PCR-) fluorescence probe technique. We then performed a correlation analysis of the function, proliferation, and apoptosis of CTLs, and we performed EBNA-1 gene knock out using siRNA technologies to observe the influences of EBNA-1 on the CD8+ T cells of patients with SAA to clarify the roles of EBV in the immune pathogenesis of SAA.

## 2. Methods

### 2.1. Study Participants

Twenty-seven patients with SAA (17 male, 10 female; median age, 32 years; age range, 9 to 68 years) were enrolled in this study. Fourteen patients with a new diagnosis who had not yet undergone immunosuppressive therapy were placed in the untreated SAA group (nine male, five female; median age, 35 years; age range, 14 to 68 years), and 13 patients were enrolled in the SAA remission group (eight male; five female; median age, 32 years; age range, 9 to 61 years). The patients' diagnoses were all in accordance with the following diagnostic criteria [14, 15]. (1) The degree of bone marrow cell proliferation was less than 25% of normal; if between 25% and 50%, the proliferation level of the remaining hematopoietic cells had to be less than 30%. (2) Two or three of the following parameters were met: a neutrophil count of less than 0.5 × 10^9^/L, a platelet count of less than 20 × 10^9^/L, and a reticulocyte count of less than 20 × 10^9^/L. The participants were screened for paroxysmal nocturnal hemoglobinuria, myelodysplastic syndromes, myelofibrosis, and Fanconi anemia. Patients were excluded if they had complications such as iron overload, malignancy, other autoimmune diseases, or pregnancy. All patients underwent immunosuppressive therapy, which mainly comprised rabbit antithymocyte globulin (ATG Genzyme, Polyclonals S.A.S., France), cyclosporine, and hematopoietic stimulating factors (including erythropoietin, granulocyte colony-stimulating factor, thyroid peroxidase, and interleukin IL-11). Complete remission was defined as a neutrophil count of greater than 0.5 × 10^9^/L, a platelet count of at least 100 × 10^9^/L, and a hemoglobin level up to 110 g/L. Partial remission was defined as a hemogram amelioration after immunosuppressive therapy but without achieving the standards for complete remission [16]. The SAA remission group in our study included patients with complete remission and patients with partial remission.

Ten healthy controls (three male, seven female) with a median age of 24.5 years (range, 21 to 62 years) were enrolled in this study. The study was approved by the Ethics Committee of the Tianjin Medical University, China. Informed written consent was obtained from all patients or their guardians in accordance with the Declaration of Helsinki.

### 2.2. Immunomagnetic Separation of CD8+ T Cells

**Figure 1:**
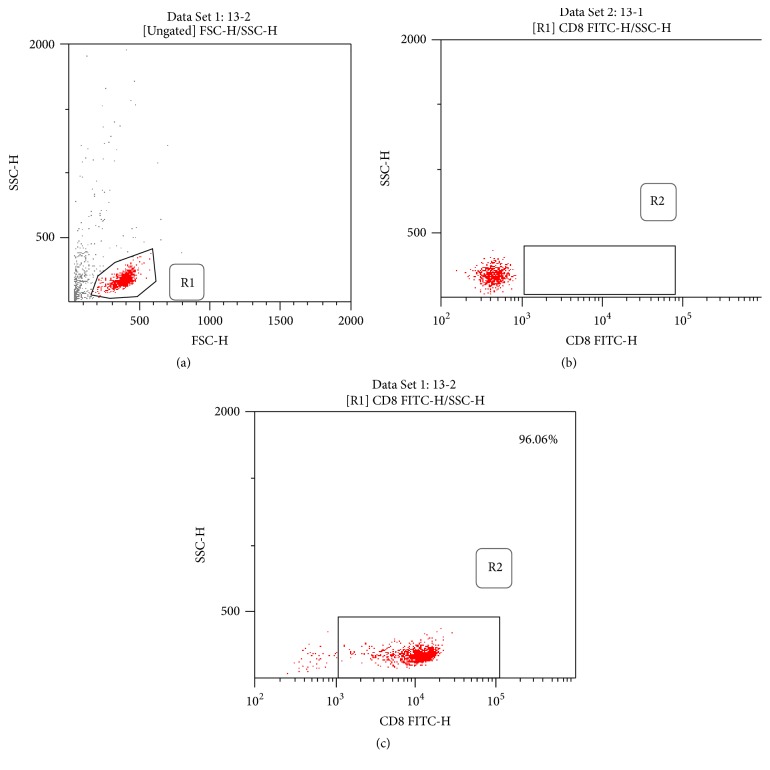
The isolated CD8+ T cells were analyzed by FCM. (a) Gating information. (b) CD8 depleted negative control sample. (c) CD8+ T cells sorting form SAA patients. The purities of isolated CD8+ T cells were 92%–97%.

A 20 ml sample of peripheral blood was collected in ethylenediaminetetraacetic acid (EDTA) anticoagulated tubes and diluted with an equal volume of phosphate-buffered saline solution (PBS). Peripheral blood mononuclear cells were then isolated from blood by gradient centrifugation using Ficoll-Paque (Amersham Bioscience, Uppsala, Sweden) and counted under a microscope with a counting plate. CD8+ T cells were then gradually sorted according to the instructions of CD8 immunomagnetic beads (Miltenyi Biotech, Germany), after which we detected the purity of sorting cells with a fluorescence-activated cell sorter- (FACS-) Calibur flow cytometer (BD Biosciences, USA) and analyzed the data with Cell Quest software (v3.1; BD Company, USA). The purity of the CD8+ T cells detected by flow cytometry was 92% to 97% (Figure 1).

### 2.3. Detection of EBV DNA Level in CD8+ T Cells with a PCR-Fluorescence Probe

Total DNA was extracted from 1 × 10^6^ sorted CD8+ T cells, and a quantitative diagnostic kit for EBV DNA (Beijing SinoMDgene Technology Co., China) was used to acquire the DNA copy numbers of the EBV BamH1W gene via PCR-fluorescence probe reaction.

### 2.4. Detection of CD8+ T Cells Functional Molecules: Perforin and Granzyme B with a Flow Cytometer

Fresh peripheral blood specimens were collected in EDTA anticoagulant tubes (100 *μ*l in each tube), to which 20 *μ*l of CD3, CD8, and their corresponding mouse isotype control (BD Company) was added. After incubation in the dark for 30 minutes at 4°C, the cells were further incubated with 2 ml of erythrocyte lytic solution (BD Pharmingen, USA) for 10 minutes at room temperature and washed with PBS, followed by permeabilization using a BD Intrasure Kit (BD Company). The cells were then stained with anti-perforin and anti-granzyme B monoclonal antibodies and their isotype control (BD Company) in the dark for 30 minutes at 4°C before washing three times with PBS. In the end, a minimum of 10,000 cells were acquired on an FACS-Calibur flow cytometer and analyzed with Cell Quest software v3.1.

### 2.5. Apoptosis Measurement of CD8+ T Cells with a Flow Cytometer

Fresh peripheral blood specimens were collected in EDTA anticoagulant tubes (100 *μ*l in each tube), and Annexin V: fluorescein isothiocyanate apoptosis detection kit I (BD Company) was used to measure the apoptosis of CD8+ T cells with an FACS-Calibur flow cytometer. The data were then analyzed by Cell Quest software.

### 2.6. Cell Viability Test of CD8+ T Cells with a Cell Counting Kit (CCK8)

The sorted CD8+ T cells were collected and resuspended using serum-free medium before inoculation into 96-well plates with 3 × 10^3^ cells in 100 *μ*l in each well; meanwhile, an equal volume of serum-free medium was taken into the plates as blank holes. After incubation for 2 hours at 37°C and 5% CO_2_ in the presence of 10 *μ*l of CCK8 (Beyotime Biotechnology, China) in each well, the absorbance at 450 nm was determined via enzyme-linked immunosorbent assay (ELX800 BioTek, USA). Cell viability was determined as the experimental well absorbance minus the blank well absorbance.

### 2.7. Detection of Serum Interferon *γ* and IL-4 by ELISA

Fresh peripheral whole blood (5 ml) was collected in EDTA anticoagulant tubes, and serum was obtained by centrifugation for 30 minutes and stored at −80°C for cytokine analysis. An interferon (IFN) *γ* and IL-4 ELISA detection kit (Cloud-Clone, USA) were applied to determine the concentration of IFN-*γ* and IL-4.

### 2.8. Experiment In Vitro

#### 2.8.1. Knockdown EBNA-1 Gene of SAA Patients with EBNA-1-siRNA

The sterile sorted CD8+ T cells of SAA patients were collected and resuspended using serum-free medium before inoculation into 24-well plates with 2.5 × 10^5^ cells in each well, and a matched blank hole was set up as a control for each test hole. 10 samples were randomly selected for the further analysis. EBNA-1-siRNA or a negative control (siRNA; GenePharma, China) was mixed with Lipofectamine 2000 (Invitrogen, USA) and added to the test hole and the control hole separately; we then discarded the supernatant after incubation for 4 hours and resuspended with complete medium containing 10% fetal bovine serum before incubation for 48 hours at 37°C and 5% CO_2_.

#### 2.8.2. Determination of the EBNA-1 mRNA Expression before and after EBNA-1-siRNA Knockdown by Q-PCR

Total RNA was extracted from the transfected CD8+ T cells using TRIzol reagent and reversed transcribed into cDNA using a TIANScript RT Kit (Tiangen, Beijing, China). The sequences of primers specific for EBNA-1 (forward: 5′-CGC TCC TAC CTG CAA TAT CA-3; reverse: 5′-CCA GGG AGG CAA ATC TAC TC-3′) and GAPDH (forward: 5′-GGT GAA GGT CGG AGT CAA CGG A-3′; reverse: 5′-GTC ATG GAT GAC CTT GGC CAG G-3′) were designed by Huada Biotech (Shanghai, China). Quantitative reverse transcription polymerase chain reaction (qRT-PCR) was performed with a Bio-Rad iQ5 Real-Time System (Bio-Rad, USA). The amplification conditions were 95°C for 30 seconds for 1 cycle and 95°C for 5 seconds and 60°C for 34 seconds for 40 cycles. The levels of EBNA-1 mRNA were calculated using the 2^−ΔΔCt^ method ((Ct_(EBNA-1)_ − Ct_(GAPDH)_)_sample_ − (Ct_(EBNA-1)_ − Ct_(GAPDH)_)_normal_).

#### 2.8.3. Differences in Function, Apoptosis, and Cell Viability of CTLs after Knockdown EBNA-1 Gene

The EBNA-1 knockdown CD8+ T cells were collected to determine their perforin and granzyme B expression levels and apoptosis, and the viability of the CD8+ T cells was also tested by CCK8. In addition, the supernatant was obtained by centrifugation and stored at −80°C, and repeated freezing and thawing were avoided. The IFN-*γ* and IL-4 levels were determined.

### 2.9. Statistical Analysis

All statistical analyses were performed using SPSS 19.0 software. The data are presented as mean ± standard deviation (SD) for normally distributed data. Comparisons between two independent samples were done by* t*-test, and paired* t*-tests were performed for paired data. For skewed distribution, the median is presented and analyzed using the rank-sum test. Chi-square testing was adopted to compare the constituent ratio. Spearman's rank correlation test was used for correlated data. A *p* value of less than 0.05 was considered to indicate statistical significance.

## 3. Results

### 3.1. Expression Level of EBV BamH1W Sequence in CD8+ T Cells of Patients with SAA

We detected DNA copies of EBV BamH1W sequence in the CD8+ T cells of 27 patients with SAA and 10 healthy volunteers. The DNA copies were positive for 12 of the 14 patients with untreated SAA (85.71%), a significantly higher rate than in the remission group (7 of 13, 53.85%) or the normal control group (4 of 10, 40%; *p* < 0.05). The number of DNA copies of EBV BamH1W sequence in CD8+ T cells in the patients with untreated SAA (mean, 7.45 × 10^4^; range, 1.00 × 10^3^ to 9.80 × 10^6^) was obviously higher than that in the remission group (mean, 1.00 × 10^4^; range, 1.00 × 10^3^ to 1.80 × 10^6^) or the normal control group (mean, 2.32 × 10^4^; range, 1.00 × 10^3^ to 3.80 × 10^5^; *p* < 0.05), whereas no marked difference was seen in the latter two groups (*p* > 0.05; Table 1).

### 3.2. Correlation between Expression Level of EBV BamH1W Sequence in CD8+ T Cells and Their Function, Apoptosis Level, and Cell Viability in Patients with SAA

**Figure 2:**
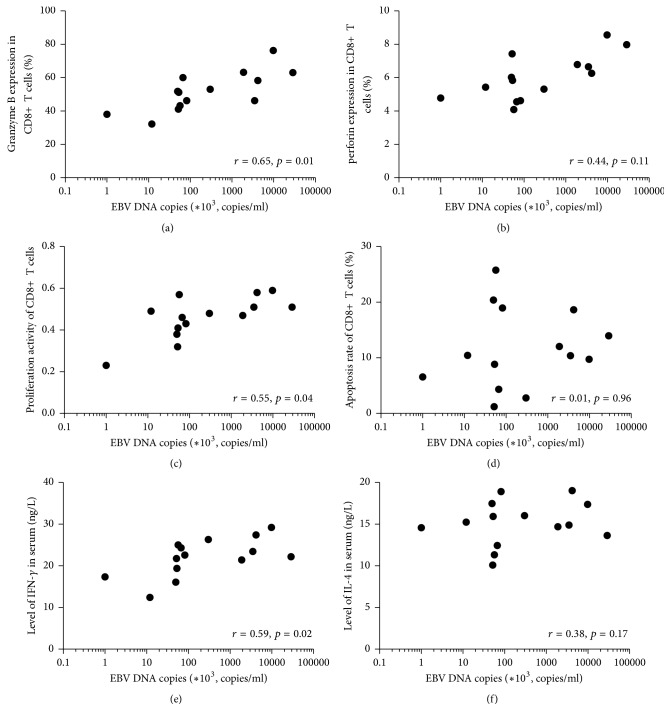
Correlation of EBV DNA copies with function, apoptosis, and proliferation of CD8+ T cells in patients with SAA untreated (*n* = 14). (a) Correlation of EBV DNA copies with Granzyme B expression in CD8+ T cells in patients with SAA untreated. (b) Correlation of EBV DNA copies with perforin expression in CD8+ T cells in patients with SAA untreated. (c) Correlation of EBV DNA copies with proliferation activity of CD8+ T cells in patients with SAA untreated. (d) Correlation of EBV DNA copies with apoptosis rate of CD8+ T cells in patients with SAA untreated. (e) Correlation of EBV DNA copies with level of IFN-*γ* in serum of SAA untreated patients. (f) Correlation of EBV DNA copies with level of IL-4 in serum of SAA untreated patients.

We also examined the expression of perforin and granzyme B in CTLs and their apoptosis, cell viability, and serum levels of IFN-*γ* and IL-4 in 14 patients with untreated SAA and analyzed their correlation with the level of EBV BamH1W DNA sequence. The results showed that the copy numbers of the EBV BamH1W DNA sequences were positively correlated with the expression of granzyme B (*r* = 0.65, *p* = 0.01), perforin (*r* = 0.61, *p* = 0.02), cell viability (*r* = 0.55, *p* = 0.04) of CTLs, and serum level of IFN-*γ* (*r* = 0.59, *p* = 0.02); however, no distinct correlation was seen between the copy numbers and apoptosis of CD8+ T cells (*r* = 0.01, *p* = 0.96) nor the level of serum IL-4 (*r* = 0.38, *p* = 0.17; Figure 2).

### 3.3. Experiments* In Vitro* to Validate the Effects of EBNA-1 on Function, Apoptosis, and Cell Viability of CTLs in Patients with SAA

#### 3.3.1. Detection of EBNA-1 mRNA Expression Level by Q-PCR before and after EBNA-1-siRNA Knockdown EBNA-1 Gene

To further illuminate the effects of EBNA-1 on CTLs in patients with SAA, we isolated the CD8+ T cells of 10 patients with untreated SAA and transfected them with EBNA-1-siRNA; we then analyzed the function, apoptosis, and cell viability of the CD8+ T cells. After transfection, we detected the EBNA-1 mRNA expression level in the CD8+ T cells using Q-PCR. The results showed that the expression level of EBNA-1mRNA in the EBNA-1-siRNA transfection group was evidently lower than that in the siRNA-negative control group (1.63 ± 0.24 versus 2.21 ± 0.31), and the difference was statistically significant (*p* < 0.05; Table 2).

#### 3.3.2. Effect of EBNA-1 Gene Knockdown on Function of CTL Cells in Patients with SAA

**Figure 3:**
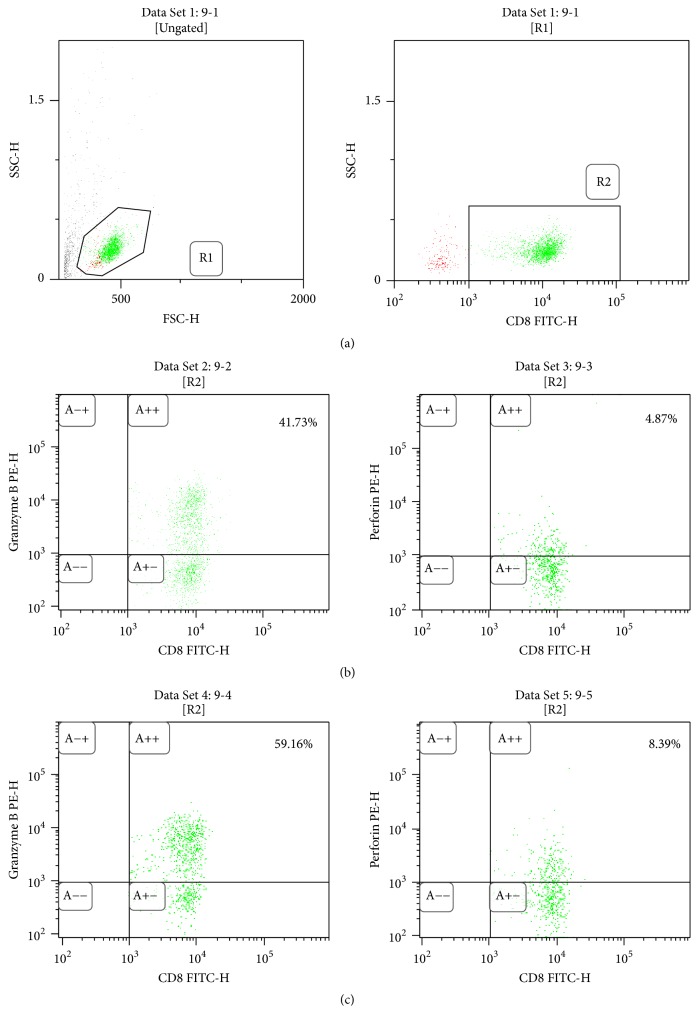
Alteration of CD8+ T cells function after silencing EBNA-1 gene. Compared with the siRNA-negative control group, the expression of Granzyme B and perforin in CD8+ T cells were decreased significantly after silencing the EBNA-1 gene (*n* = 10). (a) Gating information. (b) EBNA-1-siRNA group. (c) siRNA-negative control group.

After knockdown of the EBNA-1 gene, the expression of granzyme B and perforin in CD8+ T cells and the supernatant IFN-*γ* were all significantly lower than in the siRNA-negative control group (48.32 ± 7.10% versus 56.42 ± 8.79%, 5.26 ± 1.11% versus 6.42 ± 1.39%, and 17.13 ± 4.35 ng/L versus 20.73 ± 5.21 ng/L, resp.; *p* < 0.05). Nevertheless, no great disparity was seen in the level of the supernatant IL-4 between the EBNA-1-siRNA transfection group and the siRNA-negative control group (14.47 ± 3.48 ng/L versus 14.64 ± 2.66 ng/L; *p* > 0.05; Figure 3 and Table 3).

#### 3.3.3. Influence of EBNA-1 Gene Knockdown on Cell Viability and Apoptosis of CTLs in Patients with SAA

**Figure 4:**
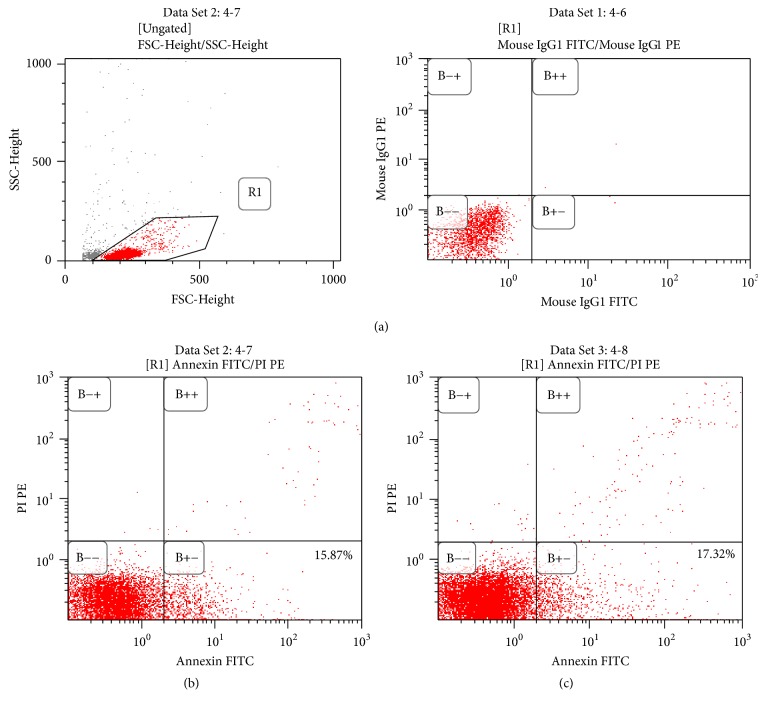
Influence of silent EBNA-1 gene on the apoptosis of CTLs in SAA patients. After silencing the EBNA-1 gene, the apoptosis rate of CD8+ T cells had no marked change comparing with siRNA-negative control group (*n* = 10). (a) Gating information. (b) EBNA-1-siRNA group. (c) siRNA-negative control group.

After EBNA-1 gene knockdown, the viability of the CD8+ T cells (0.41 ± 0.10) was apparently lower than that in the siRNA-negative control group (0.47 ± 0.07; *p* < 0.05). However, apoptosis of CD8+ T cells in the EBNA-1-siRNA transfection group showed no marked change from the siRNA-negative control group (13.78 ± 5.99% versus 12.75 ± 4.96%; *p* > 0.05; Figure 4 and Table 3).

## 4. Discussion

SAA is a kind of bone marrow hematopoietic failure caused by various factors like bone marrow infiltration and bone marrow fibrosis. Typical clinical manifestations of SAA include severe anemia, infections, bleeding, and a high risk of death. The pathogenesis of SAA is internationally recognized as an attack by autoreactive cytotoxic T cells upon bone marrow hematopoietic stem/progenitor cells. Genetics may also play a role in the pathogenesis of SAA. Studies have even considered the participation of some environmental factors (damage induced by chemicals, drugs, viruses, or antigens) in the pathogenesis of SAA by activating lymphocytes or other immune responses, especially viral infection, which has attracted considerable recent attention from scholars [17–20].

The EBV is a human B lymphocyte infectious herpesvirus that was discovered by Epstein et al. in a 1964 study of malignant lymphoma in African children. EBV infection is prevalent in many parts of the world. The EBV infection rate in normal Chinese individuals approaches 90% [21]. Patients with autoimmune diseases such as systemic lupus erythematosus and rheumatoid arthritis have a higher EBV infection rate, which has been reported frequently in recent years [22–24]. Once it has infected a human host, EBV usually lurks in the B lymphocytes and remains quiescent, but they are sometimes reactivated and begin to replicate. The immune dysfunction and lack of normal immunity in patients with AIDS causes failure of effective control of EBV infection, leading to a persistent infection or inducing reactivation. The immune regulation of EBV is mainly mediated by T cells. Once infected, antigen peptides combine with MHC1 molecules and are expressed on the surface of target cells [25]. Under auxiliary adhesion molecules, the antigen polypeptide can be recognized and combined by T cell receptor on the surface of CTLS, which gradually induces the production of toxic protein that ultimately kills the target cells. EBV can induce the secretion of IL-1, IL-6, and TNF-*α* by monocytes; contribute to the production and maturation of dendritic cells; and promote the proliferation of virus-specific CTLs [26, 27]. EBNA-1 is the only nuclear protein that is expressed in all infected cells. It is closely related to latent EBV infection and acts as the basic structure to maintain the virus genome that connects the cell chromosome as an additional form and allows this form to persist. In addition, EBNA-1 has the ability to launch the replication of EBV DNA and activate the transcription of corresponding protein encoded by EBV [28]. Furthermore, EBNA-1 can affect the interaction between ubiquitination substrates and proteasome, which further interferes with antigen processing and presentation and is intimately connected with immune escape. Embedment of the EBNA-1 gene can affect the proliferation and transformation of infected cells by adjusting the nuclear factor *κ*b, TGF-*β*, and STAT signaling pathways [29–31]. SAA caused by EBV infection has been reported in recent years, so we began a retrospective statistical study of EBV infection in all patients with treatment-naive SAA in our department and found that almost all of the patients with SAA had a previous or current EBV infection. We therefore conjectured that the EBV plays an important role in the immune pathogenesis of SAA.

Patients with SAA usually have an inverted ratio of CD4+ and CD8+ T cells. Reviews of years of basic studies have indicated lower expression of the *ζ* chain of T cell receptors, and CD8+ T cells possess oligoclonal or monoclonal amplification. With the koinonia of transcription factors T-bet and cytokines (such as TNF-*α* and interleukin), IFN-*γ*, as a bone marrow inhibitory cytokine (Th1 factor), plays an essential role in the pathogenesis of idiopathic AA. CD8+ T cells have been recognized by scholars internationally as the “archcriminal” in the occurrence of SAA. Hence, we tested the BamH1W DNA sequence of the EBNA-1 gene in the CD8+ T cells of patients with SAA to explore the role of EBV in the activation of CTLs in patients with SAA. Our results show that the CD8+ T cells in most patients with untreated SAA had higher EBNA-1 DNA expression, suggesting the existence of EBV DNA sequence insertion in patients with SAA; furthermore, the expression level was higher than that in normal control subjects. We also examined the function, apoptosis rate, and viability of the CD8+ T cells of patients with SAA and analyzed their correlation with the copy numbers of the EBV BamH1W DNA sequence. The results showed that the copy numbers of the EBV BamH1W DNA sequence were positively correlated with the granzyme B, perforin, and IFN-*γ* levels. Moreover, as the EBNA-1 DNA level rose, the viability of CD8+ T cells showed a significant synchronous increase, whereas no changes were seen in their apoptosis rate. These results suggest that as the disease severity increased, the expression of the BamH1W sequence increased correspondingly. This indicates that EBV was evidently correlated with the function and viability of CD8+ T cells, suggesting that EBV has a strengthened correlation with the excessive function of CD8+ T cells and with the pathogenesis and progress of SAA.

To further authenticate the influences of EBNA-1 gene insertion on the function and proliferation of CD8+ T cells, we blocked the transcription and translation of the EBNA-1 gene using siRNA interference technology. We then evaluated the function, viability, and apoptosis rate of CD8+ T cells before and after siRNA interference. The results showed that, as functional molecules of CD8+ T cells, granzyme B and perforin had markedly lower expression after knockdown of the EBNA-1 gene. Decreased levels of IFN-*γ* secretion and cell viability of CD8+ T cells were seen, which affirmed that EBNA-1 gene insertion had a strong influence on the function of CD8+ T cells in patients with SAA patients. CD8+ T cells are key pathogenic cells of SAA, and our study showed that EBV infection is likely to be a vital factor in the activation of CD8+ T cells, further damaging the bone marrow hematopoietic function in patients with SAA.

The pathogenesis of autoimmune diseases remains unclear. Because EBV is an autoimmune disease with the hyperfunctioning of T lymphocytes, correlation between EBV infection and the pathogenesis of SAA requires further investigation. Our study preliminarily corroborates the role of EBV in the function of CD8+ T cells in patients with SAA but suggests that other relevant findings remain that will enable us to reveal the actual role of EBV in the pathogenesis of SAA. Long-term follow-up should be initiated in patients with SAA so that they can receive a corresponding antiviral treatment in a timely manner. Moreover, these results suggest that greater attention be given to the detection of EBV and the improvement of detection methods in the hope of improving the clinical therapy and prevention of SAA in the future.

## 5. Conclusions

We infer that EBV infection is a likely factor in the activation of CTLs and in damage to the bone marrow hematopoietic function of patients with SAA.

## Figures and Tables

**Table 1:** The number and positive rate of BamH1W DNA copies in CD8+ T cells in SAA untreated patients, SAA remission patients, and normal controls.

	*n*	Copies (copies/ml)	Positive rate (%)
SAA untreated	14	7.45 × 104 (1.00 × 103–9.80 × 106)^*∗*#^	85.71%^*∗*#^ (12/14)
SAA remission	13	1.00 × 104 (1.00 × 103–1.80 × 106)	53.85% (7/13)
Normal control	10	2.32 × 104 (1.00 × 103–3.80 × 105)	40.00% (4/10)

Data was presented as median. ^*∗*^Compared with SAA remission patients, *p* < 0.05. ^#^Compared with normal control, *p* < 0.05.

**Table 2:** EBNA-1 RNA levels of CD8+ T cells transfected with EBNA-1-siRNA or siRNA negative control plasmid.

	*n*	2^−ΔΔCt^EBNA-1
EBNA-1-siRNA	10	1.63 ± 0.24
siRNA negative control	10	2.21 ± 0.31
*p* value		0.001

**Table 3:** Function, apoptosis, and proliferation of CD8+ T cells transfected with EBNA-1-siRNA or siRNA negative control plasmid.

	*n*	EBNA-1-siRNA	siRNA negative control	*p* value
Granzyme B (%)	10	48.32 ± 7.10	56.42 ± 8.79	0.02
Perforin (%)	10	5.26 ± 1.11	6.42 ± 1.39	0.03
Apoptosis rate (%)	10	13.78 ± 5.99	12.75 ± 4.96	0.48
Proliferation activity	10	0.41 ± 0.10	0.47 ± 0.07	0.03
IFN-*γ* (ng/L)	10	17.13 ± 4.35	20.73 ± 5.21	0.04
IL-4 (ng/L)	10	14.47 ± 3.48	14.64 ± 2.66	0.85

## Data Availability

The data used to support the findings of this study are available from the corresponding author upon request.
